# Factors Influencing Midazolam Dose for Intravenous Sedation in Dental Patients With Anxiety: A Retrospective Observational Study

**DOI:** 10.3290/j.ohpd.c_2226

**Published:** 2025-08-26

**Authors:** Hassan Abed

**Affiliations:** a Hassan Abed Assistant Professor and Consultant of Special Care Dentistry and Conscious Sedation, Department of Basic and Clinical Oral Sciences, Faculty of Dental Medicine, Umm Al-Qura University, Makkah, Saudi Arabia. Conceptualisation, data curation, methodology, validation, formal analysis, investigation, writing – original draft, and writing – review and editing.

**Keywords:** conscious sedation, dental anxiety, dental extraction, dentistry, midazolam

## Abstract

**Purpose:**

This study aimed to assess factors that impact midazolam dose for intravenous sedation (IVS) in dental patients with anxiety.

**Materials and Methods:**

This was a retrospective, observational study for adult, anxious patients (moderate to severe dental anxiety) who had different types of dental procedures under IVS with midazolam and local anaesthesia. A logbook of dental patients who had dental procedures was used to collect data on an Excel sheet (Microsoft Excel Workbook 2024).

**Results:**

Data of 233 patients were recorded. The average dose of IVS with midazolam delivered was 6.62 mg (SD = 3.24). Multivariable logistic regression found that two variables were statistically significant predictors for the IVS with midazolam dose, which are age (B = 1.30, S.E = 0.47, Exp(B) = 3.68, 95% CI = 1.45-9.33, P = 0.006) and non-surgical periodontal therapy with root planing (B = 0.85, SE = 0.39, Exp(B) = 2.35, 95% CI = 1.08–5.12, P = 0.031).

**Conclusions:**

Younger patients and non-surgical periodontal therapy with root planing appear to be predictors for higher doses of IVS with midazolam. Other variables that were not predictors to affect IVS with midazolam dose, such as medical history, American Society of Anesthesiologists (ASA) classification, medications, and others, are crucial, and they should not be neglected when designing the treatment plan to deliver dental treatment under IVS with midazolam.

Dental anxiety is found to be among the most common reasons for reduced access to dental care,^8^ consequently worsening the patient’s oral or dental health in general. It is therefore crucial that dentists have knowledge about valid strategies to treat anxious patients in order to reduce the number of dental patients who miss their dental appointments due to high anxiety levels.^14^

Literature reported that conscious sedation techniques exhibit promising results when used to deliver different types of dentistry to anxious patients.^8^ Several conscious sedation techniques, such as inhalation sedation using nitrous oxide mixed with oxygen, intravenous sedation (IVS), oral sedation, and intranasal sedation with midazolam,^3^ can be used.

Among these approaches, IVS with midazolam is widely used to effectively treat adult patients with moderate to severe dental anxiety when they cannot accept dental procedures under local anaesthesia (LA) alone.^1,2^ This technique strongly requires clinical training and education to be applied safely and wisely by dentists.^3^ However, less is currently known about the factors that could influence the administration of IVS with midazolam dose among patients in dental clinics. Therefore, this retrospective study aimed to assess factors that impact midazolam dose for IVS in dental patients with anxiety.

## MATERIALS AND METHODS

### Study Setting, Design, and Eligibility

This was a retrospective study for adult, anxious patients (moderate to severe dental anxiety) who had different types of dental procedures under IVS with midazolam and LA. Only adult dental patients were eligible for this study. Patients are considered to have moderate to severe anxiety when they cannot accept or tolerate any dental procedure under LA alone. A logbook (maintained by a trained [HA]) of dental patients who had dental procedures at multi-secondary care centres from 2015 to 2024 was used to collect data on an Excel sheet (Microsoft Excel Workbook 2024). All patients received IVS with midazolam using one protocol as explained next.

### Measures

#### Demographic data

Age, gender, medical history and medications of the patients, mental capacity based on the Mental Capacity Act (MCA),^6^ American Society of Anesthesiologists (ASA) classification,^5^ and cannabis usage were recorded.

#### Types of dental procedures and conscious sedation details

Type of dental procedures, intravenous sedation dose, number of cases required pre-IVS, intranasal sedation (INS), number of cases requiring reversal with flumazenil and reported side effects were collected.

Ellis’s sedation score for each patient to assess quality of IVS were also collected (ie, grade I: no limb movement, excellent co-operation and no restlessness; grade II: some limb movement, excellent total co-operation and no restlessness; grade III: more limb movement with some degree of restlessness and anxiety, acceptable level of cooperative, and dentist able to perform all dental procedures; grade IV: major limb movement and head movement, poor co-operation, patient quite restless and anxious, dentists can deliver only basic dental procedures, and lastly grade V: obvious and major restlessness, anxiety and limb and head movement, and dentists unable to deliver any dental procedure).^4^

#### IVS with midazolam procedures

All patients and their escorts/caregivers were informed about the pre-sedation instructions at least 24 h before starting dental treatment, such as taking a prescribed daily medication and having a light meal prior to IVS. The availability of a responsible adult escort is mandatory before any type of dental procedure under IVS with midazolam.

Before starting dental treatment under IVS, pre-sedation vital signs were recorded (ie., non-invasive blood pressure, oxygen saturation (SpO_2_), and heart rate). Immediately after reclining the dental chair, a cannulation technique was initiated using a tourniquet to access and use available veins (mainly the antecubital fossa). The area was disinfected with chlorhexidine gluconate in an applicator (1.5 mL), then a cannula (BD Nexiva®) was easily inserted and covered using a transparent film dressing.

Craig’s (2027) regimen for IVS with midazolam was considered.^3^ For example, each patient was injected with 2 mg (2 mL) of midazolam over 30 s; paused for 90 s, and an additional 1 mg increment was added every 30 s until a good and acceptable level of sedation was reached. It is important to make sure that the oxygen saturation is not less than 90%.

Once the patients were well sedated, profound LA was given based on the area of treatment and the type of dental procedures (ie, infiltration versus block). Patient was considered sedated when he/she showed signs of relaxation, opened his/her mouth without resistance, and responded to commands easily. All patients had their clinical evaluation regularly during dental treatment under IVS with midazolam, assessing their skin complexion, quality of consciousness, and breathing (depth and rate). The overall average duration of the dental procedure was 60 min or less. For patients who underwent longer dental treatment (>60 min), a higher dose of midazolam than the average was administered, if the patients needed it.

For the recovery, all patients were discharged under the care of the responsible adult escorts/caregivers when they were able to walk independently, their vital signs returned to normal measures, their cannula was removed, and their wound was dressed. Postoperative and IVS instructions were given to the patients and their escorts/caregivers (verbal and written). The administration of flumazenil post-IVS with midazolam was needed for special care patients with challenging behaviour to make their journey home easy.

### Statistical Analysis

Categorical and nominal variables are presented as counts and percentages, and continuous variables as means and standard deviations. Chi-square test was used to assess the statistically statistically significant association between the categorical variables. A binary logistic regression analysis with backwards conditional elimination (entry criterion: 0.05, removal criterion: 0.10) was used to identify statistically significant predictors for IVS with midazolam dose (dependent variables) at a 95% confidence level. A P value of < 0.05 was considered statistically statistically significant using IBM SPSS version 27 (IBM, Armonk, NY, USA).

### Sample Size Calculation

The theory of event per variable of 10 regarding the sample size calculation of the logistic regression analysis was used [number of independent variables × 10].^10^ Accordingly, a minimum number of 120 patients (60 per subgroup) is enough to assess which variables might be considered as a predictor for the IVS with midazolam dose at 5% level of significance.

### Ethical Consideration

Written consent was obtained from all patients as a part of a routine step before any dental procedure under conscious sedation. Data collected and the subject patients were kept in a secure folder with utmost confidentiality.

## RESULTS

### Demographic Characteristics

Table 1 presents demographic details of the patients (n = 233). The majority of the patients were female (n = 135, 57.9%) with an average age of 41.21 (SD = 16.0), having maintained mental capacity (n = 218, 93.6%), and had not used cannabis (n = 221, 94.8%). Roughly one-third of them were fit and well (n = 76, 32.6%). On the ASA classification, nearly half of them were healthy patients (ASA-I) (n = 95, 40.8%), and around one-third had mild systemic diseases (ASA-II) (n = 81, 734.8%).

**Table 1:** Demographic characteristics

Demographics	N	Min	Max	Mean	SD
	Count	%
Age	233	13	81	41.21	16.0
Total	233	100.0
Age	<=30	73	31.3
31–45	76	32.6
46–60	53	22.7
>60	31	13.3
Gender	Male	98	42.1
Female	135	57.9
Medical Hx	Fit and well	76	32.6
Cardiovascular diseases	31	13.3
Head and Neck cancer	2	0.9
Blood disorder	8	3.4
Hema-oncological diseases	5	2.1
Psychological disorders	15	6.4
Craniofacial syndromes	9	3.9
GIT disorders	3	1.3
Musculoskeletal disorders	6	2.6
Respiratory diseases	22	9.4
Autoimmune diseases	9	3.9
Learning disability	23	9.9
Skin cancers	2	0.9
Neurological disorders	17	7.3
Endocrine diseases	5	2.1
ASA	I – Normal healthy patients	95	40.8
II – Mild systematic diseases	81	34.8
III – Severe systematic diseases that are limiting but not incapacitating	52	22.3
IV – Severe incapacitating disease that is a constant threat to life	5	2.1
Mental capacity	Maintained mental capacity	218	93.6
Lacking mental capacity	15	6.4
Cannabis	Yes	12	5.2
No	221	94.8
Medication	ACE inhibitors	6	2.6
Anti-coagulant	3	1.3
Anti-depressant	8	3.4
Anti-diabetic	2	0.9
Anti-platelets	6	2.6
Anti-psychosis	2	0.9
Anti-seizure	8	3.4
B-blocker	5	2.1
Biological agent	21	9.0
Bisphosphonate	1	0.4
Bronchodilator	19	8.2
Calcium channel blocker	5	2.1
CBD	14	6.0
Chemotherapy	7	3.0
Corticosteroid	10	4.3
Diazepam	3	1.3
Diclofenac	1	0.4
Folic acid	2	0.9
Gabapentin	5	2.1
Iron supplement	2	0.9
Methotrexate	4	1.7
Nil	79	33.9
NSAID	6	2.6
Oral contraceptive	2	0.9
Protien pump inhibitor	3	1.3
Radio-chemotherapy	2	0.9
Thiazide	3	1.3
Thyroxin supplement	2	0.9
Warfarin	2	0.9


### Types of Dental Procedures and Conscious Sedation Details

Table 2 presents the types of dental procedures delivered and the conscious sedation details of 233 patients. Most patients had more than 5 mg of intravenous sedation, with an average dose of IVS with midazolam delivered of 6.624 mg. Only 21 patients had pre-IVS intranasal (5.2%), and 23 patients used flumazenil (9.9%) to reverse the effect of sedative drugs. Most patients were cooperative and had an excellent quality of sedation (Grade I) on the Ellis sedation score (n = 214, 91.8%). The delivered dental procedures were mostly dental restoration (n = 107, 45.9%), followed by dental extraction (n = 59, 25.3%) and non-surgical periodontal therapy with root planing (n = 57, 24.5%), with very few cases of dental examination alone (n = 10, 4.3%). Very few reported side effects were noticed, such as body movement, coughing, dysphagia, crying, head movement, screaming, seizure, and talkative episodes (n = 21, 9%).

**Table 2:** Types of dental procedures and conscious sedation details

Variables	N	Min	Max	Mean	SD
	Count	%
Intravenous sedation dose	233	0.5	20.0	6.62	3.24
Total	233	100.0
Dental procedure	Dental examination	10	4.3
Non-surgical periodontal therapy	57	24.5
Dental restoration	107	45.9
Dental extraction	59	25.3
Intravenous sedation dose	< = 5	113	48.5
>5	120	51.5
Ellis score	Grade I	214	91.8
Grade II	14	6.0
Grade III	5	2.1
Pre-intranasal sedation dose		21	5.2
Flumazenil		23	9.9
Reported side effects	None	212	91.0
Body movement	6	2.6
Coughing	1	0.4
Coughing and dysphagia	3	1.3
Crying	1	0.4
Head movement	5	2.1
Screaming	1	0.4
Seizure	2	0.9
Talkative	2	0.9


**Fig 1:**
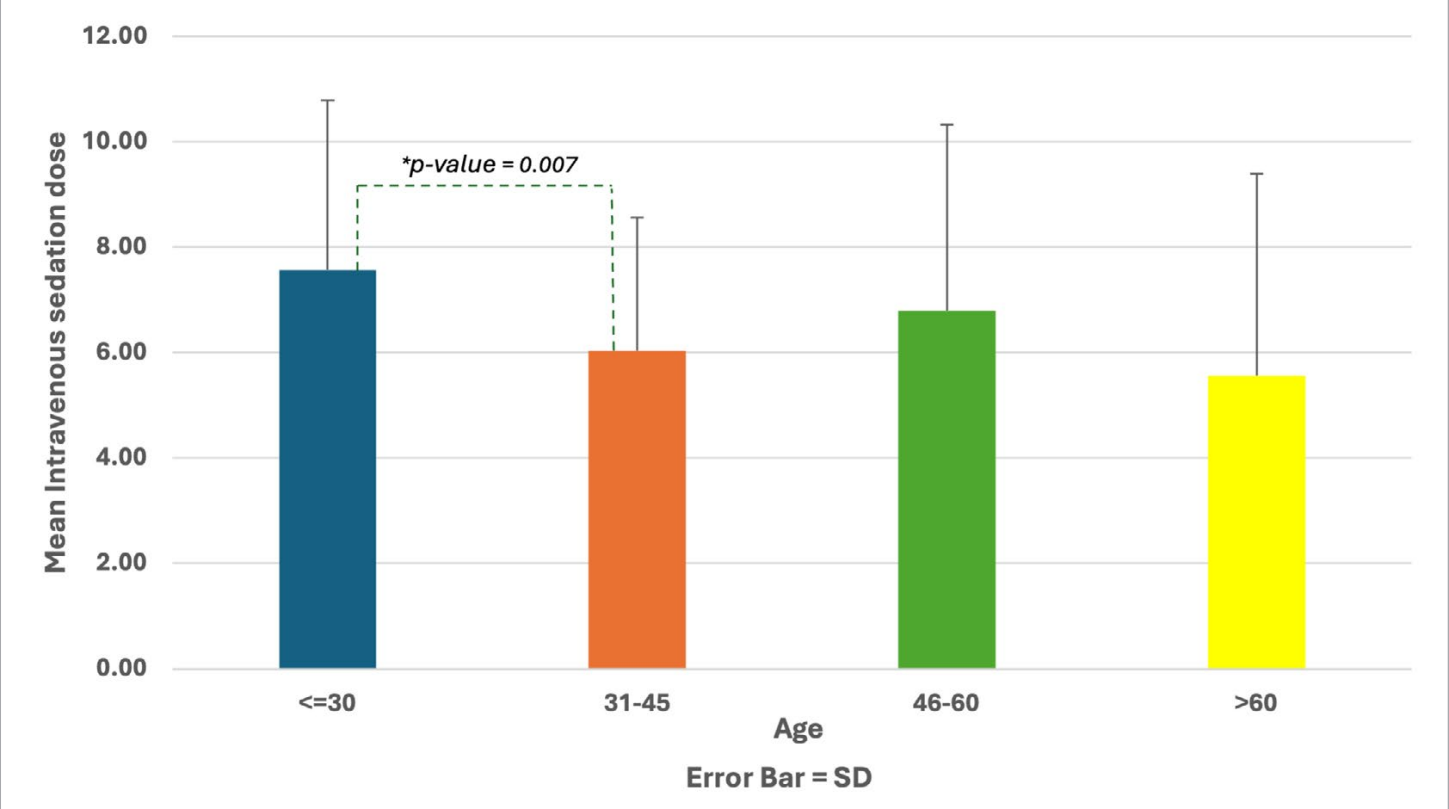
Association between intravenous sedation dose and age.

**Fig 2:**
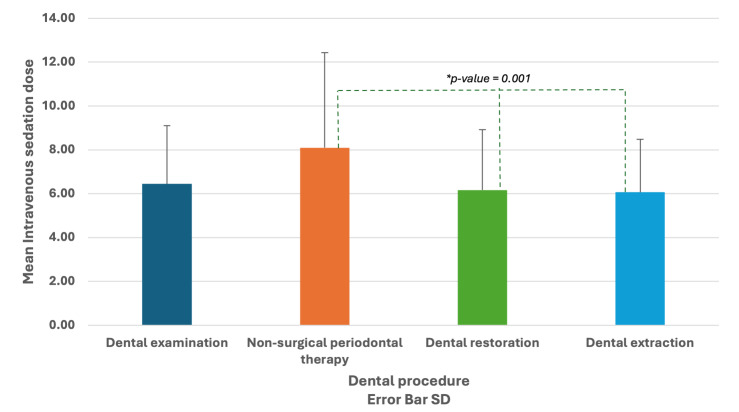
Association between intravenous sedation dose and type of dental procedures.

Chi-squared tests showed that there were statistically significant differences between the IVS with midazolam dose of patients against factors such as age and type of dental procedure delivered. Younger patients (<31 years old) received statistically significantly higher doses of midazolam than older patients (P = 0.007) (Fig 1). Additionally, patients who had non-surgical periodontal therapy with root planing received statistically significantly higher doses of midazolam than other types of dental procedures (P = 0.001) (Fig 2).

### Differences Between Male and Female

**Fig 3:**
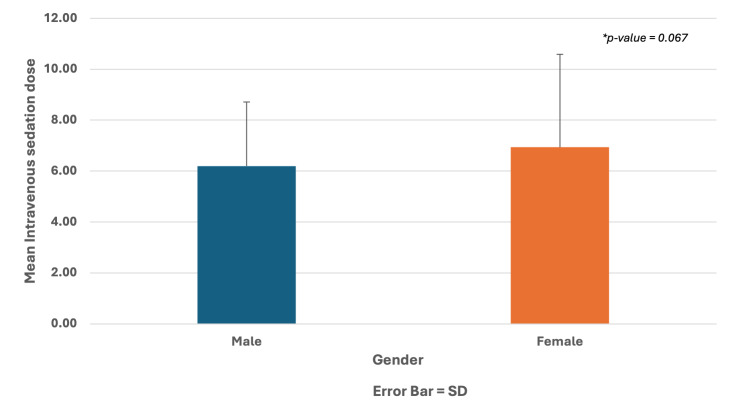
Association between intravenous sedation dose and gender.

Table 3 presents the differences between male and female patients on demographic characteristics, type of dental procedure delivered, and conscious sedation details. Chi-square tests showed that there were statistically significant differences between male and female patients on the type of medical history (P < 0.001), ASA classification (P < 0.001), and the need for the flumazenil post-IVS with midazolam (P = 0.005). On the other hand, the Chi-squared test showed that there was no statistically significant difference between the IVS with midazolam dose of patients, regardless of whether male or female (P = 0.067) (Fig 3).

**Table 3:** Differences between male and female

Variables	Total	Male	Female	P value
Total	233	98 (42.1%)	135 (57.9%)	–
Medical Hx	Fit and well	76	19 (25.0%)	57 (75.0%)	<0.001^a^
Cardiovascular diseases	31	24 (77.4%)	7 (22.6%)
Head and Neck cancer	2	1 (50.0%)	1 (50.0%)
Blood disorder	8	2 (25.0%)	6 (75.0%)
Hema-oncological diseases	5	2 (40.0%)	3 (60.0%)
Psychological disorders	15	7 (46.7%)	8 (53.3%)
Craniofacial syndromes	9	3 (33.3%)	6 (66.7%)
GIT disorders	3	0 (0.0%)	3 (100.0%)
Musculoskeletal disorders	6	3 (50.0%)	3 (50.0%)
Respiratory diseases	22	5 (22.7%)	17 (77.3%)
Autoimmune diseases	9	6 (66.7%)	3 (33.3%)
Learning disability	23	16 (69.6%)	7 (30.4%)
Skin cancers	2	0 (0.0%)	2 (100.0%)
Neurological disorders	17	7 (41.2%)	10 (58.8%)
Endocrine diseases	5	3 (60.0%)	2 (40.0%)
ASA	I – Normal healthy patients	95	31 (32.6%)	64 (67.4%)	<0.001^a^
II – Mild systematic diseases	81	48 (59.3%)	33 (40.7%)
III – Severe systematic diseases that are limiting but not incapacitating	52	19 (36.5%)	33 (63.5%)
IV – Severe incapacitating disease that is a constant threat to life	5	0 (0.0%)	5 (100.0%)
Mental capacity	Maintained mental capacity	218	92 (42.2%)	126 (57.8%)	0.867
Lacking mental capacity	15	6 (40.0%)	9 (60.0%)
Cannabis	Yes	12	7 (58.3%)	5 (41.7%)	0.241
No	221	91 (41.2%)	130 (58.8%)
Dental procedure	Dental examination	10	7 (70.0%)	3 (30.0%)	0.279
Non-surgical periodontal therapy	57	21 (36.8%)	36 (63.2%)
Dental restoration	107	45 (42.1%)	62 (57.9%)
Dental extraction	59	25 (42.4%)	34 (57.6%)
Intravenous sedation dose	< = 5	113	47 (41.6%)	66 (58.4%)	0.889
>5	120	51 (42.5%)	69 (57.5%)
Ellis score	Grade I	214	91 (42.5%)	123 (57.5%)	0.422
Grade II	14	4 (28.6%)	10 (71.4%)
Grade III	5	3 (60.0%)	2 (40.0%)
Flumazenil	Yes	23	16 (69.6%)	7 (30.4%)	0.005^a^
No	210	82 (39.0%)	128 (61.0%)
^a^ Statistically statistically significant using Chi-Square test at <0.05 level.

### Factors Impacting IVS With Midazolam Dose

Multivariable logistic regression found that two variables were statistically significant predictors for the IVS with midazolam dose, namely the young age group (≤ 30 years old) and the medical procedure of non-surgical periodontal therapy with root planing. Patients aged 30 years or younger had 1.304 times higher chances of receiving a higher IVS dose than older patients (SE = 0.47, Exp(B) = 3.68, 95% 1.45–9.33, P = 0.006). This also suggests that younger patients metabolise the sedative differently or have varying levels of anxiety or pain tolerance, thereby necessitating higher doses (Table 4).

**Table 4:** Factors affecting intravenous sedation with midazolam dose for dentistry

Variables in the equation	B	SE	Exp (B)	95% CI for EXP (B)	P value
Dependent variable: intravenous sedation dose	Lower	Upper
First step^a^	Age						0.114
< = 30	1.189	0.496	3.285	1.244	8.677	0.016^b^
31–45	0.708	0.478	2.030	0.796	5.177	0.138
46–60	0.818	0.504	2.266	0.844	6.080	0.104
Gender						
Male	0.120	0.305	1.127	0.620	2.048	0.695
ASA						0.796
I – Normal healthy patients	1.126	1.207	3.085	0.290	32.864	0.351
II – Mild systematic diseases	1.101	1.211	3.008	0.280	32.315	0.363
III – Severe systematic diseases that are limiting but not incapacitating	0.950	1.215	2.586	0.239	27.964	0.434
Mental capacity						
Maintained mental capacity	0.411	0.634	1.508	0.436	5.220	0.517
Cannabis = Yes	0.549	0.717	1.732	0.425	7.067	0.444
Dental procedure						0.006^b^
Dental examination	0.428	0.746	1.535	0.356	6.620	0.566
Non-surgical periodontal therapy	0.843	0.415	2.322	1.031	5.234	0.042^b^
Dental restoration	–0.439	0.351	0.645	0.324	1.284	0.212
Ellis score						0.863
Grade I	0.413	0.976	1.511	^0.223^	^10^.242	0.672
Grade II	0.610	1.131	1.841	0.200	16.904	0.590
Flumazenil = Yes	–0.284	0.499	0.753	0.283	2.003	0.569
Constant	–2.651	1.622	0.071			0.102
Last step^a^	Age						0.055
< = 30	1.304	0.474	3.683	1.453	9.334	0.006^b^
31–45	0.854	0.464	2.348	0.945	5.834	0.066
46–60	0.888	0.488	2.430	0.933	6.328	0.069
Dental procedure						0.005^b^
Dental examination	0.490	0.713	1.633	0.404	6.598	0.492
Non-surgical periodontal therapy	0.854	0.397	2.349	1.079	5.115	0.031^b^
Dental restoration	–0.433	0.339	0.649	0.334	1.261	0.202
Constant	–0.852	0.445	0.427			0.055
^a^ Variable(s) entered on step 1: Age, Gender, ASA, Mental capacity, Cannabis, Dental procedure, Ellis score, Flumazenil. ^b^ Statistically significant using Binary Logistic Regression Model, with Backward Conditional Elimination with Enter Criteria = 0.05, Elimination = 0.10.

Moreover, non-surgical periodontal therapy with root planing is associated with more than double the likelihood of requiring higher IVS doses (B = 0.85, SE = 0.39, Exp(B) = 2.35, 95% CI = 1.07–5.14, P = 0.031) compared to dental examination alone (B = 0.49, SE = 0.71, Exp(B) = 1.63, 95% CI = 0.40–6.59, P >0.05).

## DISCUSSION

In this study, a logistic regression model was employed to identify predictors of IVS with midazolam dose in dental procedures, specifically distinguishing between doses of 5 mg or less and those exceeding 5 mg. Multivariable logistic regression analysis found that two variables were statistically significant predictors of higher midazolam doses: age and non-surgical periodontal therapy with root planing.

Present works revealed that younger patients require higher midazolam doses than older patients. This is consistent with the systematic review and meta-analysis on the global estimated prevalence of dental anxiety, reporting dental anxiety to be high among women and younger adults.^13^ Additionally, several studies reported that young adults have high dental anxiety levels^7,9^; hence, they require higher doses to reduce their anxiety levels. Second, elderly patients have less affinity of receptors to midazolam particles^13^; hence, this group of patients requires a lower dose of midazolam when compared to younger groups of patients to reduce the risk of minimal effect of respiratory depression.^11^ Lastly, the number of areas required for non-surgical periodontal therapy with root planing is higher than that of anaesthetised areas required for a single tooth restoration or extraction.

This study also found that patients who had non-surgical periodontal therapy and root planing required higher midazolam doses than other patients who had different types of dental procedures. A cross-sectional study assessed the relationship between dental anxiety and pain perception during non-surgical periodontal therapy and root planing.^14^ They found a statistically significant association between dental anxiety and pain perception during non-surgical periodontal therapy and root planing. For example, participants (n = 100) had an average of 17.3 ± 13.8 pain on the visual analogue scale during non-surgical periodontal therapy and root planning.^12^

This study also revealed that the average dose of IVS with midazolam required to deliver dental care was 6.62 (SD = 3.24). This average dose is aligned to the most commonly suggested dose for dental procedures (usual dose = 2–7.5 mg).^3^ Indeed, titration is the practical and measured way when delivering IVS with midazolam to deliver safe dental care and reduce the risk of respiratory depression. Each dental patient has a different level of dental anxiety, previous dental experience and different thoughts regarding dentistry. Therefore, each patient should have a customised dental treatment, and their needs should be met by the midazolam dose.

### Strengths and Limitations of the Study

The strength of this study includes the utilisation of the regression model (backwards conditional elimination), allowing systematic identification of potential predictors and consequently removing the least statistically significant variables to improve the model’s parsimony. On the other hand, the study includes limitations such that the model lacks a theoretical framework, which may be prone to spurious correlations. Other limitations include possible predictors that may not be fully addressed (eg, body mass index (BMI)) and bias associated with incomplete records because of retrospective design.

## CONCLUSIONS

Younger patients and non-surgical periodontal therapy with root planing appear to be predictors for higher doses of IVS with midazolam when required to deliver dental care for anxious adult patients. Although other variables were not predictors to affect IVS with midazolam doses, such as medical history, ASA classification, medications, and others, these are crucial, and they should not be neglected when designing the treatment plan to deliver dental treatment under IVS with midazolam and should be considered to deliver safe and high-quality dental care in a relaxed environment.

### Acknowledgements

Ethical approval was obtained from the Institutional Review Board at Umm Al-Qura University (HAPO-02-K-012-2024-10-2232).

This research received no specific grant from any funding agency in the public, commercial, or not-for-profit sectors.

Thanks are extended to Fathiya Bakhsh for her assistance with this research project and data extraction.
